# The cardiovascular changes underlying a low cardiac output with exercise in patients with type 2 diabetes mellitus

**DOI:** 10.3389/fphys.2024.1294369

**Published:** 2024-03-20

**Authors:** Per Lav Madsen, Casper Sejersen, Michael Nyberg, Martin Heyn Sørensen, Ylva Hellsten, Peter Gaede, Annemie Stege Bojer

**Affiliations:** ^1^ Department Cardiology, Herlev-Gentofte Hospital, Copenhagen University, Copenhagen, Denmark; ^2^ Department Clinical Medicine, Copenhagen University, Copenhagen, Denmark; ^3^ The August Krogh Section for Human Physiology, Department Nutrition, Exercise and Sports, Copenhagen University, Copenhagen, Denmark; ^4^ Department of Anaesthesia, Rigshospitalet, Department of Clinical Medicine, University of Copenhagen, Copenhagen, Denmark; ^5^ Department Kidney and Vascular Biology, Global Drug Discovery, Novo Nordisk, Copenhagen, Denmark; ^6^ Department Endocrinology, Slagelse-Næstved Hospital, Copenhagen, Denmark

**Keywords:** type 2 diabetes mellitus, heart failure with a preserved ejection fraction, exercise intolerance, hypertension, cardiac fibrosis, myocardial blood flow and flow reserve

## Abstract

The significant morbidity and premature mortality of type 2 diabetes mellitus (T2DM) is largely associated with its cardiovascular consequences. Focus has long been on the arterial atheromatosis of DM giving rise to early stroke and myocardial infarctions, whereas less attention has been given to its non-ischemic cardiovascular consequences. Irrespective of ischemic changes, T2DM is associated with heart failure (HF) most commonly with preserved ejection fraction (HFpEF). Largely due to increasing population ages, hypertension, obesity and T2DM, HFpEF is becoming the most prevalent form of heart failure. Unfortunately, randomized controlled trials of HFpEF have largely been futile, and it now seems logical to address the important different phenotypes of HFpEF to understand their underlying pathophysiology. In the early phases, HFpEF is associated with a significantly impaired ability to increase cardiac output with exercise. The lowered cardiac output with exercise results from both cardiac and peripheral causes. T2DM is associated with left ventricular (LV) diastolic dysfunction based on LV hypertrophy with myocardial disperse fibrosis and significantly impaired ability for myocardial blood flow increments with exercise. T2DM is also associated with impaired ability for skeletal muscle vasodilation during exercise, and as is the case in the myocardium, such changes may be related to vascular rarefaction. The present review discusses the underlying phenotypical changes of the heart and peripheral vascular system and their importance for an adequate increase in cardiac output. Since many of the described cardiovascular changes with T2DM must be considered difficult to change if fully developed, it is suggested that patients with T2DM are early evaluated with respect to their cardiovascular compromise.

## Introduction

The significant morbidity and premature mortality of type 2 diabetes mellitus (T2DM) is largely associated with its cardiovascular consequences. Focus has long been on the arterial atheromatosis of DM giving rise to early stroke and myocardial infarctions, whereas less attention has been given to its non-ischemic cardiovascular consequences. In 1954, the Danish endocrinologist Knud Lundbæk was the first to document arterial wall changes associated with DM *per se*, and in 1969 he suggested that DM may be associated with heart disease even if un-accompanied by coronary atheromatosis. In the early 1970′s, studies of patients (pts.) with DM, who had died from heart failure (HF) revealed an association with vast islands of myocardial fibrosis despite normal coronary arteries. These findings proved Lundbæk right and confirmed the existence of a non-ischemic “diabetic cardiomyopathy” (Rubler et al., 1972). While DM may be associated with dilated non-ischemic cardiomyopathy, i.e., HF with reduced ejection fraction (HFrEF), T2DM in general gives rise to HF with preserved ejection fraction (HFpEF; Lav Madsen, 2022). Secondary to increasing age and high prevalence of obesity, hypertension, and T2DM, HFpEF is now the dominant form of HF worldwide, and the prevalence is expected to rise (Borlaug et al., 2023a). Years of randomized controlled studies (RCTs) of HFpEF treatment, with pts. stratified mainly on echo-cardiographically determined cardiac diastolic movement patterns, have been largely futile (Borlaug et al., 2023a), and it now seems logical to turn to the specific phenotypes of HFpEF, i.e., if HFpEF-symptoms are based on, for example, amyloidosis or diabetic cardiomyopathy. Weight-loss through glucagon like peptide (GLP) 1 receptor analogues will improve exercise capacity and lower natriuretic peptide levels in patients with obesity (Borlaug et al., 2023b; Kosiborod et al., 2023), but besides this no treatments for frank HFpEF exists except for limited symptom alleviation with sodium-glucose cotransporter-2 inhibitors (SGLT2i) and (possibly) spironolactone (Borlaug et al., 2023a). Medicinal regulatory authorities increasingly accept that new medication for HFpEF can now be approved based on symptom-improvement, since in pts. with HFpEF dyspnea and impaired exercise capacity can be so devastating that pts. favor improved life-quality as much as prolonged longevity. The scarcity of effective therapies for HFpEF emphasizes the importance of identifying the underlying phenotypes, and distinct pathophysiological mechanisms, if meaningful interventions to test in RCTs are to be found. It is now recognized that once frank HF is diagnosed in T2DM, the phenotypical changes leading to HF have developed over years and will be challenging to reverse. Consequently, there is growing interest in studying early stages of HFpEF, referred to as “pre-HFpEF” (Gori et al., 2021). In pts. with T2DM, this may mean already at the time diagnosis of T2DM before overt and irreversible exercise intolerance occurs.

In this review, cardiovascular changes with T2DM, among the most prevalent structural reason for HFpEF, both cardiac and peripheral changes are reviewed with the aim of addressing the major cardio-physiological systems implicated in the ability to provide for a high enough cardiac output (CO) to honor the whole-body oxygen uptake (VO_2_) needed for an active life without overt breathlessness. Changes with T2DM are compared with findings in the well-trained (that will be used to describe the normal biological changes seen with a systematically increased VO_2_ demand), and findings from pts. with obesity and hypertension are included to the extend they shed light on findings in pts. with T2DM, since obesity, hypertension, and T2DM often go hand-in-hand (hence the sometimes-used overarching terms “diabesity” and “metabolic syndrome”).

## The HFpEF syndrome in T2DM

The syndrome of HFpEF is an overarching heterogenous “umbrella” term, describing exercise incapacity, overt dyspnea, and pulmonary congestion in pts. with a seemingly normal heart function. Recent reviews have highlighted major gaps in our knowledge of its causes, but reviews often remain “cardio-centric,” with little emphasis on peripheral cardiovascular causes. In T2DM, the hypertrophic “stiff” left ventricle (LV) with increased wall thickness (Mohan et al., 2015) and the associated impaired filling and increased end-diastolic pressure remains a well-documented cause of HFpEF (“diastolic dysfunction”; Abudiab et al., 2013; Borlaug et al., 2023a), but several complementary pathophysiologic mechanisms exist including abnormal ventricular-arterial coupling with equally “stiff” peripheral circulations have been documented (Shibata et al., 2011; Nesti et al., 2020; Kyhl et al., 2021). Also, integrative cardiovascular studies have documented detrimental changes to the peripheral circulation with DM, but such changes remain to be incorporated into clinical perspectives.

Numerous studies have documented lower VO_2max_ during exercise in pts. with T2DM in comparison with age-matched controls. The VO_2max_ values are usually 20%–30% lower than in controls (Nesti et al., 2020), although the magnitude can vary depending on the control group studied. Initially, the reduced VO_2max_ is a consequence of a sedentary lifestyle (Hamilton et al., 2007). With time and progression of T2DM, however, significant and possibly irreversible cardiovascular changes will pose their own limit to the ability to exercise, and even in well-regulated pts. with T2DM, a reduction in the efficiency with which oxygen is converted to external work has been demonstrated (reduced slope of the VO_2_/workload relationship during graded exercise; Regensteiner et al., 1998). Frank and Starling at the turn of the 20th Century demonstrated the heart to be “permissive” and that the main function of the heart is to forward-deliver the blood provided from the systemic veins (Frank, 1895; Frank, 1898; Patterson et al., 1914; Patterson and Starling, 1914; Starling and Visscher, 1926). In line, Guyton demonstrated that the prevailing CO results from an integration of venous return with cardiac function (Guyton et al., 1957). It is mainly when the heart cannot forward-deliver the venous blood returned to it (or can only do so with increased end-diastolic pressure) that the term HF should be applied, but long before that, pts. can have had exercise incapacity from lowered venous return by impaired skeletal muscle vasodilation.

## Cardiac output changes with exercise in patients with T2DM

In normal subjects, at the onset of exercise, heart rate (HR) and ventricular stroke volume (SV) increase thereby allowing CO to closely match the metabolic demand of the working skeletal muscles (Higginbotham et al., 1986; Mortensen et al., 2005). In normal subjects, CO increases from ∼5 L/min at rest to ∼15 L/min at maximal whole body exercise in young females (Wang et al., 2012) and ∼20 L/min in young males (Mortensen et al., 2008) and up to ∼25–30 L/min (Wang et al., 2012) and ∼35–40 L/min (Ekblom and Hermansen, 1968) in elite female and male athletes. The increase in HR is responsible for most of this CO augmentation, but as HR is not increased by training, the large increase in exercise CO of the well-trained is the result of a larger SV (Bada et al., 2012; Munch et al., 2014). SV rises during exercise as a result of increases in LV end-diastolic volume and, to a lesser extent, sympathetically mediated reduction in LV end-systolic volume. LV end-diastolic volume is determined by diastolic filling, which is determined by a complex interplay between HR, intrinsic myocardial relaxation, ventricular compliance, ventricular filling pressures, atrial contraction and pericardial and pulmonary constraints (Baggish and Wood, 2011). Accordingly, increased volumes of the heart chambers are now well-established hallmarks of the “athlete’s heart” (Pelliccia et al., 1996). While overall few differences are found in resting systolic and diastolic LV function among trained athletes as compared to sedentary controls (Pluim et al., 2000), it seems LV diastolic function in exercise is enhanced by prolonged systematic training (Levine, 2008).

**FIGURE 1:**
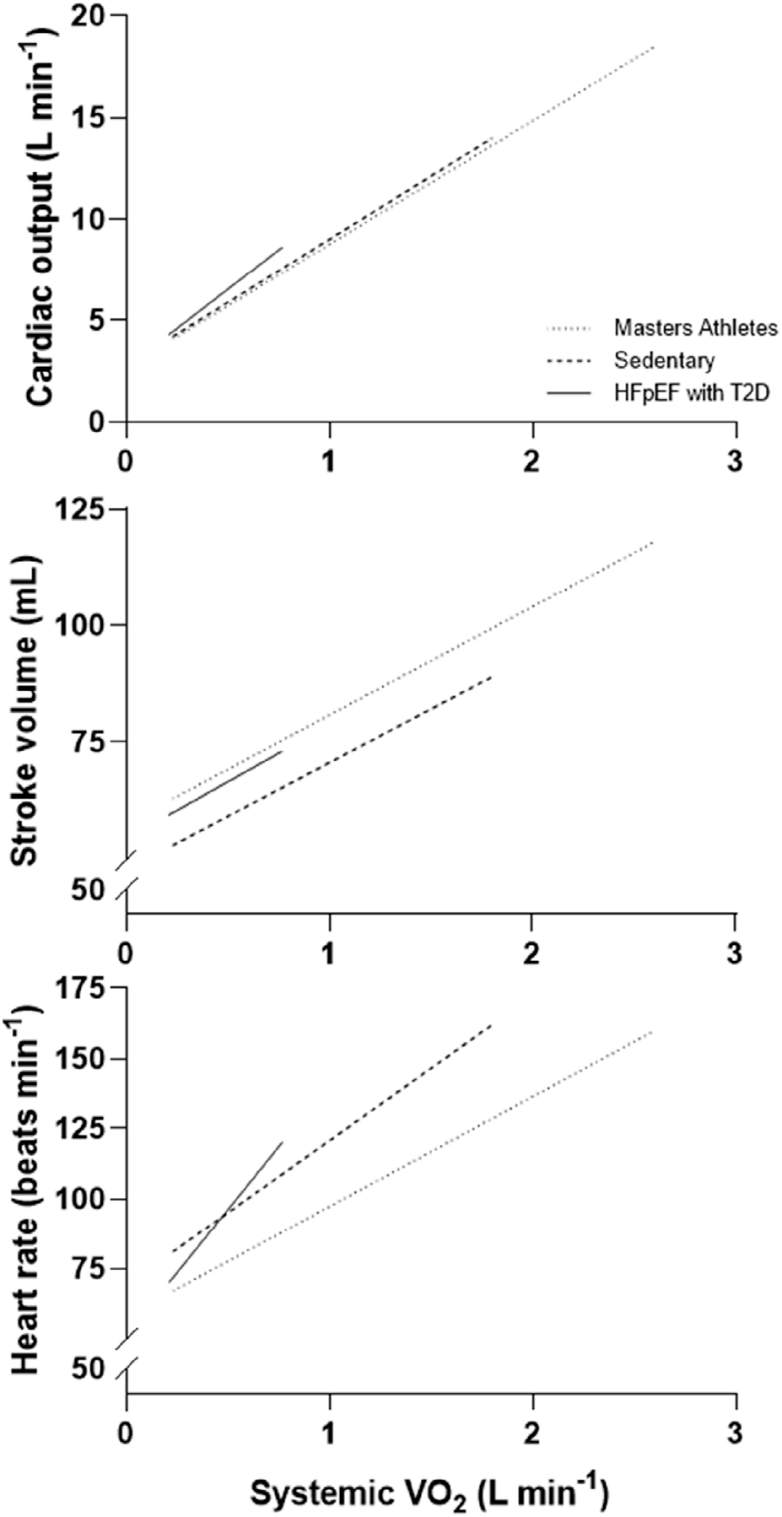
Cardiac output, stroke volume and heart rate in relation to systemic oxygen uptake (VO_2_) in patients with heart failure with preserved ejection fraction and T2DM vs. age-matched untrained and master athletes (example with representative values from various published sources). The same amount of needed daily work to–say - walk to the near-by grocery are performed by the athlete and the sedentary obese patient with T2DM in two markedly different ways: For the well-trained athlete, the systemic VO_2_ required for this task will only be a fraction of what she or he can maximally deliver, and will be delivered with a high stroke volume and only need for a very small increase in heart rate, probably a heart rate increase that will only encompass a lowered vagal tone on the heart, and hardly any need for further increments in heart rate from increased sympathetic drive. For the sedentary subjects and notably for patients with HFpEF and type 2 diabetes, the systemic VO_2_ required is much closer to sub-maximal work, and will in these be performed with only a small or negligent increase in cardiac stroke volume but a considerably higher heart rate, - a heart rate that will be much closer to the maximally attainable heart rate, especially so if chronotropic incompetence has occurred.

In pts. with T2DM, the CO increase during exercise is lower than in normal age-matched subjects (Kim et al., 2015; Figure 1). Together with notably a smaller SV, but also a lower HR at comparable workloads, the increase in CO is easily attenuated by ∼20% that is also matched by a ∼20% lower maximal workload in comparison with controls (Pinto et al., 2014; Kim et al., 2015). Even without differences in LV end-systolic volumes, during sub-maximal exercise LV end-diastolic volume is low in pts. with DM in comparison with normal age-matched controls (Wilson et al., 2017; Roberts et al., 2018). Albeit not yet studied in pure T2DM populations, it is generally observed that the common finding is that a CO increase in CO with exercise is severely restricted in pts. with frank HFpEF. When compared to normal subjects, CO is blunted in relation to the obtained VO_2max_, with 20% lower CO-to-VO_2_ slope seen in pts. with HFpEF than in age-matched normal subjects (Abudiab et al., 2013). The significantly reduced VO_2max_ in HFpEF pts. is predominantly attributable to the CO limitation, as the HFpEF pts.’ low VO_2max_ is coupled with blunted increases in HR (Keytsman et al., 2015), SV as well as LVEF (Abudiab et al., 2013). In one study, age-matched normal subjects increased CO to 13 vs. only 9 L/min in the pts. with severe HFpEF. In frank HFpEF, the CO increase with exercise is therefore easily 50% reduced in comparison to what is normally seen in even untrained young healthy adults (Abudiab et al., 2013).

## Cardiac causes of low CO during exercise in patients with T2DM

### Left ventricular hypertrophy and impairment in T2DM

Abundant echocardiographic studies demonstrate that LV hypertrophy is a notable consequence of T2DM, and that this hypertrophy is an independent predictor of adverse cardiovascular outcomes including HF (Dawson et al., 2005; Gosse, 2005). The arterial hypertension seen in seven out of 10 pts. with T2DM increases afterload to the heart and hence induces hypertrophy, but while regression of LV hypertrophy with antihypertensive treatment reduces cardiovascular morbidity and mortality, insufficient regression of LV hypertrophy is often seen (Gerdts et al., 2008; de Simone et al., 2009; de Simone et al., 2013; Lønnebakken et al., 2017) and indeed LV mass increases for other reasons than explained by mere myocyte hypertrophy. Insufficient regression of LV hypertrophy is associated with a 1.7 increased hazard ratio of cardiovascular events within the next 7 years (Lønnebakken et al., 2017). The LV hypertrophy of pts. with T2DM is associated with minor signs of both systolic and diastolic impairment even at rest. The most important cardiac changes predictive of future HFpEF, are discrete signs of systolic impairment (from global longitudinal strain) and signs of diastolic dysfunction with impaired e` (early diastolic longitudinal relaxation velocity of the left ventricle) and increased E/e’ (blood velocity during early diastolic inflow to the left ventricle as compared to the e’; Jørgensen et al., 2019). With a normal E/e’ <8, frank HFpEF is associated with values > 12–14, and many pts. with T2DM will have values in-between. Patients with T2DM therefore develop small chambered thick-walled hearts that are in many aspects virtually the antithesis to athletes’ hearts with increased chamber volumes.

### Left ventricular fibrosis in T2DM

**FIGURE 2:**
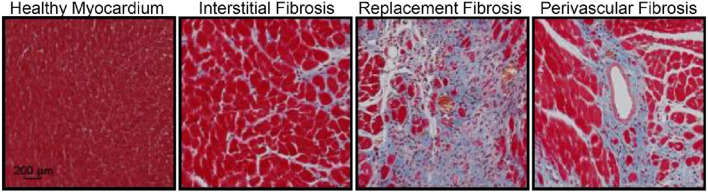
The three different aspects of fibrotic myocardial changes (Masson’s trichrome staining of the mouse heart demonstrating fibrosis (blue) in the healthy myocardium) not exclusively but often seen in diabetes (from Thomas and Grisanti, 2020). These changes are in patients with type 2 diabetes non-invasively determined with cardiac magnetic resonance imaging from the extracellular volume fraction (increasing with diffuse interstitial fibrosis), gadolinium late-enhancement imaging (depicting areas of non-ischaemic replacement fibrosis), and the myocardial perfusion ratio (decreasing with perivascular fibrosis).

The information that HFpEF in T2DM relates to the heart having hypertrophied and “stiffened” with subtle signs of systolic and diastolic dysfunction does not help much for the selection of future intervention goals, if the mechanisms which underlie these alterations are not understood. It has been convincingly demonstrated in rodents, but now also demonstrated in humans from recent cardiac magnetic resonance imaging scans, that the initial hypertrophy (often inward giving rise to small LV cavities) seen in T2DM cannot be solely explained by myocyte hypertrophy from increased afterload but is also due to the expansion of the myocardial extracellular volume (ECV) from fibrosis (Sørensen et al., 2020a; Sørensen et al., 2020b; Sørensen et al., 2020c; Bojer et al., 2020; Bojer et al., 2021a; Bojer et al., 2021b; Bojer et al., 2022; Bojer et al., 2023; Lav Madsen, 2022). In pts. with T2DM, the increase in extracellular volume is the consequence of interstitially situated advanced glycation end-products cross-linking abundant collagen micro-fibrils (Russo and Frangogiannis, 2016), and hence determination of the ECV is an imaging biomarker of disperse fibrosis. The formation of advanced glycation end-products is not only seen in the myocardium but also in vascular walls (Gonzalez et al., 2018; Frangogiannis, 2021). Perivascular fibrosis provides for “tight sheets” around the myocardial arterioles; whereas the more diffuse fibrosis that develops later becomes dispersed between cardiac myocytes (Gonzalez et al., 2018; Frangogiannis, 2021; Figure 2). In coronary arteries of pts. with ischemic heart disease including pts. with T2DM, nitric oxide production switches to hydrogen peroxide production lowering flow-mediated vasodilation (Juguilon et al., 2022). Additionally, and perhaps even more important, the perivascular fibrosis that results in vascular rarefaction, and as shown from combined endomyocardial biopsy and angiography perivascular fibrosis vascular rarefaction, perivascular fibrosis is responsible for microvascular dysfunction, i.e., lowering of myocardial blood flow during stress (Dai et al., 2012). Of interest for future clinical target determination, the perivascular fibrosis is linked to expression of mRNA signaling in pathways related to both fibrosis and osteochondrosis, whereas interstitial fibrosis is mainly related to expression of mRNA signaling related to fibrosis (Thisted et al., 2021). A detailed account of the reasons for cardiac fibrosis in T2DM is beyond the scope of this review, but in short, increased fibrosis has been attributed to inflammation, oxidative stress, an increase in endothelin production (Chen et al., 2000), AMP-activated kinase and notably insulin resistance and hyperglycemia (Rekhraj et al., 2013; Szwejkowski et al., 2013; Mohan et al., 2015; Russo and Frangogiannis, 2016).

**FIGURE 3:**
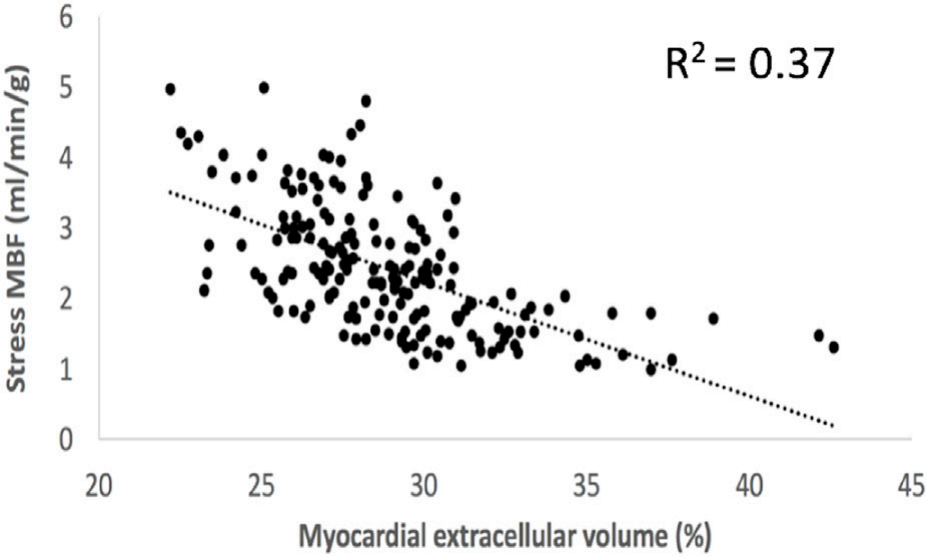
Co-variation of myocardial extracellular volume (an imaging biomarker of disperse interstitial fibrosis) and adenosine-stress induced myocardial blood flow (an imaging biomarker of perivascular fibrosis and the accompanying vascular rarefaction) in patients with type 2 diabetes (Sørensen et al., 2020c). With abundant diffuse fibrosis resulting in an extracellular volume >30%–32% also stress myocardial blood flow is significantly lowered and cannot increase much with stress.

Cardiac magnetic resonance imaging studies document that myocardial fibrosis and microvascular dysfunction also are prevalent in pts. with T2DM (Sørensen et al., 2020a; Sørensen et al., 2020b; Sørensen et al., 2020c; Bojer et al., 2020; Bojer et al., 2021a; Bojer et al., 2021b; Bojer et al., 2022; Bojer et al., 2023; Lav Madsen, 2022). Even in pre-HFpEF, pts. with T2DM have not only myocardial hypertrophy but also significantly lowered maximal myocardial blood flow during adenosine-stress (microvascular dysfunction); and widespread myocardial fibrosis with increased ECV. In some pts. even “islands of fibrosis” (“replacement fibrosis”) are associated with increased biomarkers related to HF (pro-ANP and pro-BNP; Sørensen et al., 2020a; Sørensen et al., 2020b; Sørensen et al., 2020c; Bojer et al., 2020; Bojer et al., 2021a; Bojer et al., 2021b; Bojer et al., 2022; Bojer et al., 2023; Lav Madsen, 2022; Figure 3). Whereas the normal myocardial perfusion reserve (MPR) is ∼5, i.e., myocardial blood flow can increase by a factor of 5 with adenosine-infusion, in pts. with T2DM, it is ∼3.5, and in some pts. little or no increase in perfusion is seen with stress (Sørensen et al., 2020a; Sørensen et al., 2020b; Sørensen et al., 2020c). In normal young subjects, the ECV is ∼25%, but increases to 26%–27% in middle age, only to increase up to >28% in pts. with T2DM without complications and to >29% in pts with complications and even 40% in some (Bojer et al., 2022; Lav Madsen, 2022; Figure 3). In a broad cohort, replacement fibrosis is noted in ∼10% of all pts. with T2DM, often situated in the basal inferolateral part of the LV (Bojer et al., 2020).

## Myocardial determinants of a stiff left ventricle in T2DM

**FIGURE 4:**
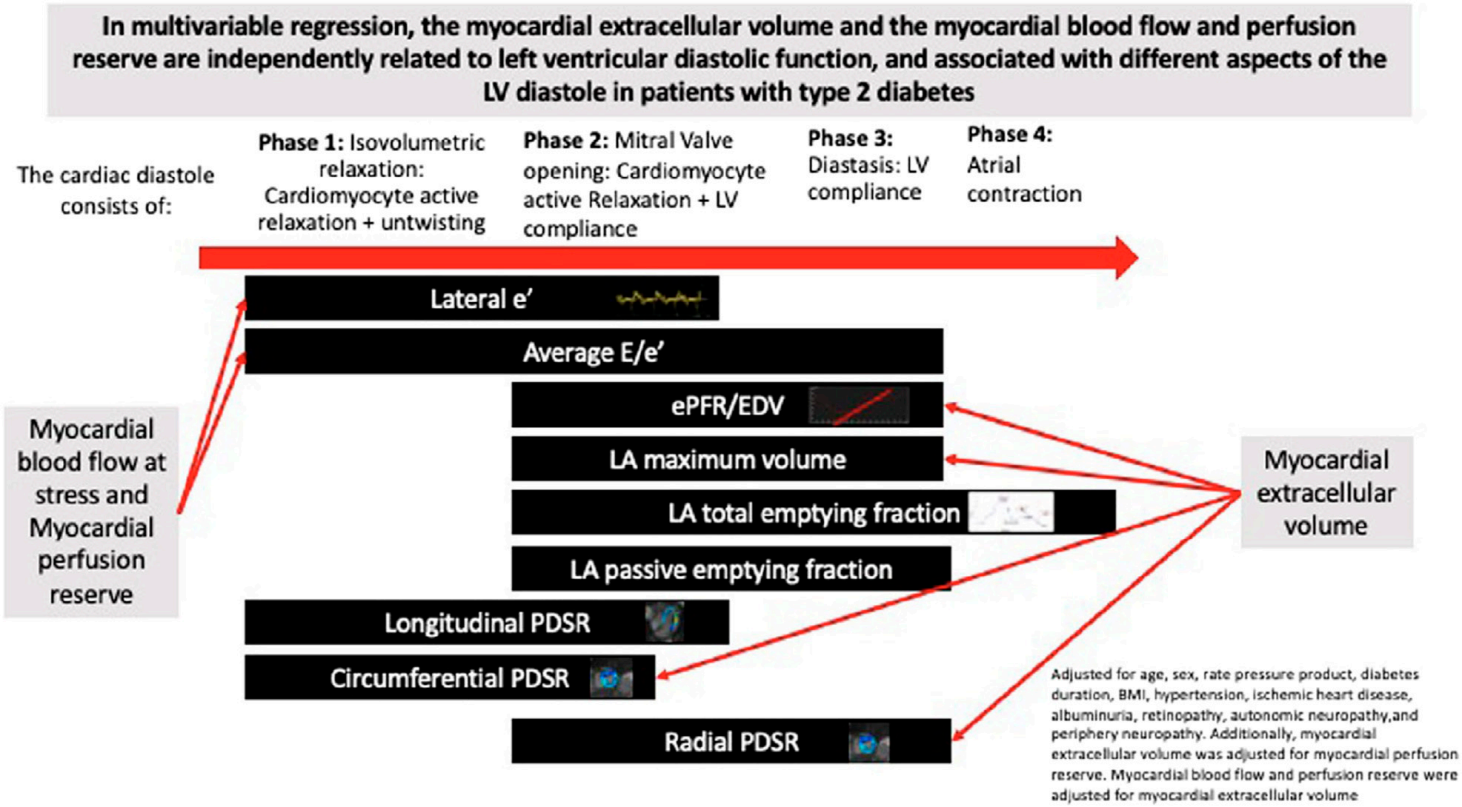
The different phases of diastole and their relation to myocardial blood flow reserve and extracellular volume (an imaging biomarker of disperse fibrosis) (from Bojer et al., 2023). In short, myocardial blood flow impairment affects early diastole (i.e., the active and adenosine triphosphate-requiring phase of diastole), whereas disperse fibrosis predominantly affects later parts of diastole (non-energy requiring).

In animal studies, diastolic dysfunction in DM largely stems from myocardial fibrosis and microvascular dysfunction with vascular rarefaction, and in diabetic animals, fibrosis impacts negatively on systolic strain parameters (Shi et al., 2022). In pts. with T2DM, we have shown that impaired MPR and ECV both negatively impacts on diastolic function at rest, among the most significant early predictors of imminent HFpEF in pts. with T2DM (Bojer et al., 2023; Bojer et al., 2024). Of interest, the different fibrosis fractions affect different aspects of diastole, as “early” (ventricular unwinding) is more affected by impaired myocardial blood flow, i.e., the perivascular fibrosis, whereas “late” (diastasis) diastolic filling of the LV is affected by an increased ECV (i.e., diffuse myocardial fibrosis; Bojer et al., 2023; Figure 4). In pts. with T2DM, an increased ECV has been associated with a reduced VO_2max_ (Jellis et al., 2011), but no studies exist on the precise impact of the different aspects of fibrosis for the risk of developing frank HFpEF. The LV fibrosis of T2DM is associated with autonomic neuropathy, concomitant ischemic heart disease and active (but not former) smoking, implicating a role for not only further anti-lipid therapy but potentially also anti-inflammatory therapies (Bojer et al., 2022). In broader pt. cohorts, where cardiac magnetic resonance imaging scans have been performed for clinical reasons, DM and increased HbA1c are independent predictors of an increased ECV, and an increased ECV independently of myocardial scars (replacement fibrosis) adds to the risk of premature HF or death (Yang et al., 2019). The recent magnetic resonance imaging studies points to more longstanding cardiac changes in T2DM as the reason for increased cardiac “stiffness” that–based on animal studies of the reason for such changes - must be considered difficult to reverse and hence to be found and treated well before overt HFpEF ensues, and it is likely that the pts. with T2DM with the worst MPR and the most prevalent fibrosis will also be the pts. that are first affected by clinically significant HFpEF.

## Myocardial determinants of a stiff left ventricle in T2DM

Not much is still known on how to reverse fibrosis of the myocardium once developed, and it is reasonable to assume that it will be difficult to fully reverse. In aged and diabetic animals, agents that can chemically break pre-existing cross-linking of collagen molecules are capable of reverting indices of vascular and myocardial compliance to levels seen in younger or non-diabetic animals, and the development of advanced glycation end-product cross-link breakers (like alagebrium) have been suggested for HFpEF in these conditions, but have not yet materialized (Fujimoto et al., 2013; Toprak and Yigitaslan, 2019). In frank HFpEF, even 1 year of intense training cannot significantly improve LV compliance and volumes, arterial stiffness, exercise capacity or ventricular-arterial coupling (Fujimoto et al., 2012), and it must be assumed that exercise at best can be used to hinder development of fibrosis. In animals, SGLT2i ameliorated myocardial oxidative stress injury and histology-verified cardiac fibrosis in diabetic mice (Li et al., 2019). Bojer et al. (2022) found a tendency for treatment with SGLT2i to lower the ECV (*p* = 0.06), and in one magnetic resonance imaging RCT, empagliflozine significantly lowered the ECV in pts. with T2DM and ischaemic heart disease (Mason et al., 2021), but it is still not known if in humans, this also reflects a lowered fibrosis content or merely a general improvement in extracellular volume content as shown in renal failure pts. (Ohara et al., 2019). Thus, these changes may be influenced by increased systemic inflammation, and although the impact of inflammation on development of fibrosis is not yet well-established, it is of interest that in pts. with obesity (but not T2DM) and signs of HF, weight-loss by GLP1 receptor agonists also improve systemic inflammation (Borlaug et al., 2023a). Mainly follow-up studies of such changes will document if treatment with SGLT2i will significantly impact on the development of HFpEF.

## Peripheral adaptations of importance to the CO-increase with exercise in T2DM

As important as it is to understand how well the heart functions in T2DM, it is no less important to study the degree of balancing of heart function with venous return. Besides the important treatment objectives for T2DM, it is therefore important to also look at cardiovascular physiological parameters that will relate to exercise tolerance.

## Blood volume changes in patients with T2DM

Of importance for exercise capacity, one notable circulatory change in DM is a limited circulating blood volume (Montero et al., 2019). In general, physical work acutely reduces plasma volume by activating the renin-angiotensin-aldosterone cascade with resultant renal water retention (Convertino et al., 1980; Convertino et al., 1981). With training, the erythrocyte volume also increases until plasma volume and the erythrocyte volume have increased by 8%–10%, with the hematocrit stabilizing at a level at or slightly below the level of sedentary subjects. The hypervolemia of athletes hence reflects a larger total body water volume with increased interstitial fluid available to sweat glands allowing for greater conductive heat exchange (Convertino, 1991; Ho et al., 1997) and a lower viscosity of the blood reducing the cardiac workload (El-Sayed et al., 2005). Further, a lower hematocrit is associated with more pronounced endothelial-derived vasodilatation (Madsen et al., 2006) probably by lesser scavenging of nitric oxide (Madsen et al., 2006; Hoiland et al., 2020). In pts. with DM, the regulation of intravascular volumes is altered by chronic activation of the renin-angiotensin-aldosterone system and the vasopressin axis (Montero et al., 2019) and from albumin-loss reducing the osmotic potential of plasma (Montero et al., 2019). Hypovolemia is hence prevalent in pts. with DM, irrespective of sex, age, and even of physical activity levels (Montero et al., 2019). Part of the difference in SV between trained and untrained has been suggested to be related to differences in blood volume (Krip et al., 1997; Bonne et al., 2014), and the lowered blood volume of pts. with T2DM may in part explain their small SV. Furthermore, the lowered blood volume may in obesity and T2DM be re-distributed away from the central circulation toward veins of the lower extremities (Ubels et al., 2001; Willenberg et al., 2010; Engelberger et al., 2014), of particular importance during upright exercise.

## Blood pressure at rest and exercise in T2DM, and its impact on cardiac function and exercise capacity

Arterial hypertension is seen in ∼70% of pts. with T2DM, and treatment with angiotensin converting enzyme inhibitors is a cornerstone of T2DM treatment (Gaede and Pedersen, 2004). A detailed account of the reasons for hypertension in T2DM is beyond the scope of this review, however, its independent impact on cardiovascular function and its relation to HFpEF in T2DM must be addressed. If endured for years, hypertension will by way of increased cardiac afterload lead to cardiac hypertrophy probably exacerbating fibrotic cardiac changes seen with T2DM. While hypertension-research commonly has focused on resting or 24-h ambulatory blood pressure, recently exercise-induced hypertension with a maximal blood pressure (usually defined as >210 mmHg in men and >190 mmHg in women) and increased blood pressure at sub-maximal exercise have been demonstrated to have independent prognostic importance (Schultz et al., 2013). Exercise hypertension is notably prevalent in pts. with T2DM (Schultz et al., 2022) and reflects baroreflex dysfunction in pts. with metabolic syndrome even without known hypertension (Dutra-Marques et al., 2021). In general, resting hypertension can be managed not only by antihypertensive medication but also by aerobic exercise (Cornelissen and Fagard, 2005; Börjesson et al., 2016). The mechanisms underlying the effect of endurance training on resting blood pressure remain undisclosed, but are likely to include vascular remodeling (Thijssen et al., 2012); changes in the renin-angiotensin system (Hsueh and Wyne, 2011); reduced sympathetic nervous activity; and enhanced function of the nitric oxide and the prostanoid systems (Nyberg et al., 2012a; Nyberg et al., 2012b; Gunnarsson et al., 2020a; Gunnarsson et al., 2020b). The effect of training on muscle sympathetic nervous system output could be related to alternations in both central command and the pressor response originating from the contracting skeletal muscle (Mitchell et al., 1983). The vasoconstrictive effect of sympathetic activity can also be reduced in the trained muscle with a period of exercise training, via reduced alpha-adrenergic receptor sensitivity to noradrenalin (Mortensen et al., 2014). Both endurance and strength training can lower resting and exercise-induced blood pressure (Saco-Ledo et al., 2022; Correia et al., 2023). Exercise training leads to a reduction in blood pressure and sympathetic drive during acute submaximal exercise performed at the same absolute intensity when muscles of the lower limb are recruited (Cousineau et al., 1977; Winder et al., 1978; Fisher and White, 1999; Ray, 1999; Rush and Aultman, 2008; Mortensen et al., 2013). Correct antihypertensive medication of ambulatory hypertension is well-established, but it is still debated which medication best relieves exercise hypertension (Chant et al., 2018).

## Conduit artery remodeling

### Conduit artery physiology in healthy subjects

Conduit and resistance arteries allow for transport of blood from the heart to the peripheral organs and the diameter and elastic properties of these arteries is dimensioned so as not to limit the blood flowing from the heart, and the proximal conduit arteries exert an important pulse smoothening role (Windkessel function, please see below). The functional consequence of an increase in arterial diameter is reduced resistance during high levels of perfusion, as changes in wall thickness and wall composition have implications for vascular compliance. It is the changes in hemodynamic forces, including shear stress and transmural pressure, that lead to remodeling of arteries and arterioles, even in the heart (Aalkjaer et al., 1987; Brown, 2003). While static exercise training increases the wall thickness of conductance arteries, probably secondary to the often-significant increments in blood pressure (Scharhag et al., 2002), endurance-trained individuals have more compliant arteries (Green et al., 2017) with reduced arterial wall thickness and increased lumen diameter (Mulvany et al., 1978; Gunduz et al., 2011). Whereas heavy resistance training may decrease arterial compliance and hence the Windkessel function, the functional consequences of endurance training are accordingly the opposite (Mohiaddin et al., 1989; Cameron and Dart, 1994; Kingwell et al., 1997).

### Conduit artery remodeling in T2DM

**FIGURE 5:**
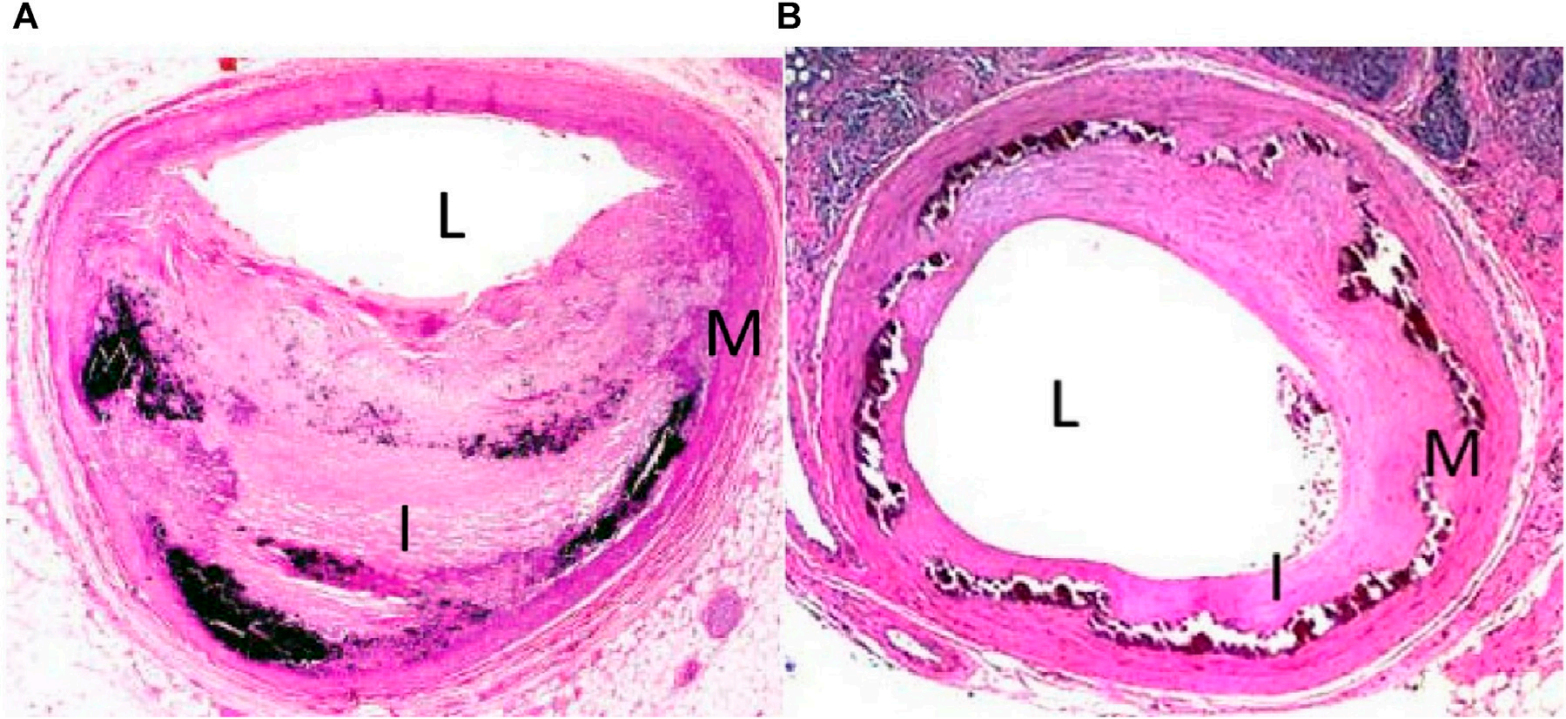
Hematoxylin and eosin stain of tibial arteries from a patient with intimal **(A)** and medial **(B)** calcification. L denotes lumen, I denotes intima, and M denotes media (from Kim and Guzman, 2023). Patients with diabesity often suffer from intimal atheromatosis, but notably patients with T2DM also often suffer from medial calcification stiffening conductance arteries and increasing afterload to the heart.

In pts. with metabolic syndrome and notably longer standing T2DM, conductance arteries including the aorta stiffen partly due to atherosclerosis, partly to media sclerosis. DM is associated not only with more narrow coronary arteries, but also with stiffening of the peripheral conductance arteries with notably smaller diameters (Frangogiannis, 2021). A more chronic increase in blood pressure likely has a greater impact on arterial wall thickness than the transient increases that occur during exercise (Gunduz et al., 2011). In media sclerosis, collagen elasticity is lost with a consequent reduction in arterial compliance. In advanced stages, large calcifications may induce secondary changes in the intima such as subendothelial hyperplasia characterized by an increase in cellularity (e.g., myofibroblasts, fibroblasts, fibrocytes) and ulcerations characterized by infiltrations of the intima or even protrusions into the lumen (Lanzer et al., 2021); (Figure 5). Such decreases in conductance including coronary arteries are associated with media sclerosis and periarterial (and periarteriolar) fibrosis with upregulation of mRNA related to not only fibrotic but also osteochondrotic pathways (Thisted et al., 2021). Media vascular wall myocytes can be differentiated into fibroblasts and thereby increase fibrosis formation and calcification (Lanzer et al., 2021). In DM, deposit of advanced glycemic end-products may also add to stiffening of conductance arteries (Fishman et al., 2018). While specific treatment modalities including statins are well-proven to improve atheromatosis, little is known about how to improve media sclerosis (Lanzer et al., 2021). Calcium-phosphate overload is important in chronic kidney disease, but in disorders with preserved systemic calcium-phosphate homeostasis, as is the case in DM, no significant specific therapy targets have been established (Lanzer et al., 2021).

**FIGURE 6:**
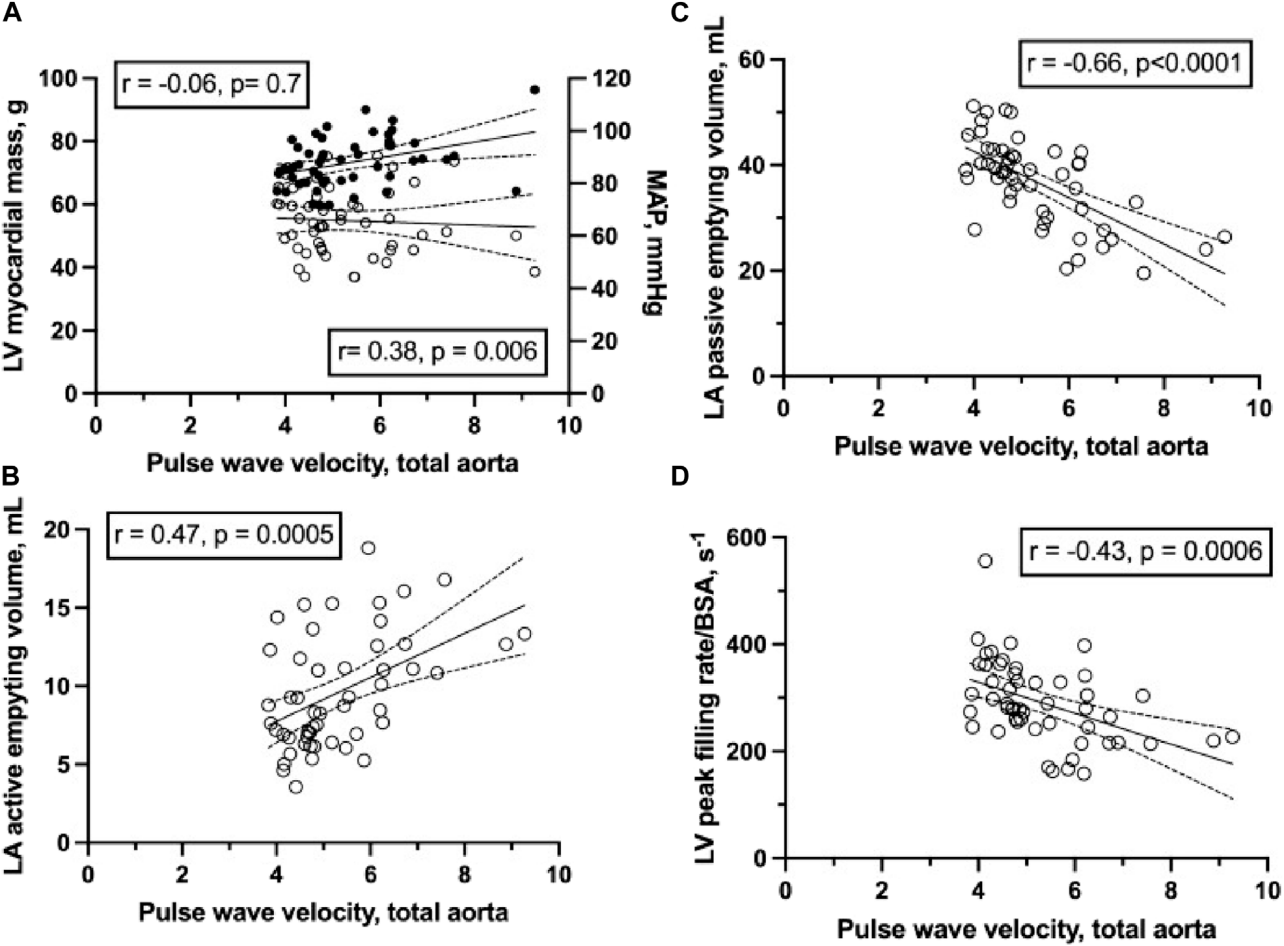
**(A)** left ventricular (LV) myocardial mass (circles, left axis) and mean arterial pressure (MAP; solid circles, right axis) to total aortic pulse wave velocity (circle) as determined from magnetic resonance imaging determined time-volume curves in 36 healthy young and 16 healthy middle-aged subjects. **(B)** left atrium (LA) active emptying volume to total aortic pulse wave velocity. **(C)** LA passive emptying volume to total aortic pulse wave velocity. **(D)** indexed LV peak filling rate to total aortic pulse wave velocity. Total aortic pulse wave velocity is correlated to LV peak filling rate (*r* = − 0.47) and both LA passive (*r* = − 0.66) and active emptying volumes (*r* = 0.47) (all *p* < 0.05) (from Kyhl et al., 2021). In short, it seems that in normal subjects stiff conductance arteries are associated with not only impaired ventriculo-arterial coupling but also impaired atrio-ventricular coupling.

The proximal conductance arterial tree (the ascending aorta, the aortic arch, and the proximal descending aorta) is important not only for unhindered forward-delivery of blood, but also for smoothing of the forward pressure wave (i.e., turning the pulsatile circulation into a somewhat lesser pulsatile system; “windkessel function”). Age and arterial blood pressure accounts for ∼50% of pulse-wave velocity, as the pulse-wave of the blood increases from normal values of 4–5 m/s to perhaps >9 m/s (Kyhl et al., 2021; Figure 6). Further stiffening of the proximal conductance arteries will be seen with abundant media calcification (McEniery et al., 2009) or aortic wall deposition of advanced glycation end-products (Semba et al., 2009), both abundant in diabesity. High pulse-waves will back-reflect from divisions of larger conductance arteries and eventually provide for further increments of left ventricular afterload/stress than imposed upon the heart from the mean arterial pressure itself. This results in not only ventriculo-arterial but also atrio-ventricular dysfunction (Kyhl et al., 2021; Figure 6). An increased aortic pulse-wave velocity is not simply due to a large burden of atheromas as demonstrated in a cohort of autopsy cases (Sawabe et al., 2005). From animal studies and prospective studies with multiple regression analysis, it is already well-known that arterial stiffness increases left ventricular afterload (Urschel et al., 1968; Milnor, 1975), myocardial work (Kelly et al., 1992), impairs ventricular relaxation (Hori et al., 1985; Kohno et al., 1996), and causes myocardial ischemia (Buckberg et al., 1972; Watanabe et al., 1993; Kaess et al., 2012). A non-distensible arterial tree results in left ventricular remodeling and ultimately dysfunction (Girerd et al., 1991; Saba et al., 1993; London et al., 2004; Zieman et al., 2005; Dai et al., 2015; Chirinos et al., 2019) and is associated with an increased risk of HF and overall cardiovascular-related mortality (Levy et al., 1990; Madhavan et al., 1994; Franklin et al., 1997; Domanski et al., 1999; Haider et al., 2003). Pts. with DM show stiff conductance arteries at an early age (Christoforidis et al., 2022), and is seen in pts. with metabolic syndrome before presenting with T2DM (Donley et al., 2014).

### Exercise as treatment for conduit artery stiffness

Aerobic exercise training reduces arterial stiffness (Donley et al., 2014), and in a controlled trial of moderate vs. high-intensity exercise, pts. with T2DM both training schemes improved carotid artery media thickness, but only the high intensity training scheme was effective in improving conductance artery stiffness (Magalhaes et al., 2019). Exercise training also reduces arterial stiffness in adults with hypertension (Lopes et al., 2021). Even in elderly pts. with DM and at risk of leg ulcers, 3 months of three weekly resistance exercise training bouts may improve signs of peripheral artery stiffness (Gholami et al., 2021). SGLT2i were early shown to lower not only blood sugars but also advanced glycation end-product formation in vessels (Shen et al., 2012) and indeed lower indices of arterial stiffness (Chilton et al., 2015; Davies et al., 2017; Pfeifer et al., 2017).

## Skeletal muscle blood flow responses to maximal and submaximal exercise

### Healthy subjects

To accommodate an increased demand for O_2_ during exercise by contracting muscle, trained individuals have a higher maximal perfusion capacity than untrained individuals, as blood flow to skeletal muscle in elite athletes can increase almost 100-fold from rest (Richardson et al., 1993; Blomstrand et al., 1997). This elevation in blood flow is achieved by an increased CO in combination with an enhancement in vascular conductance in the active muscle. Exercise hyperemia is a complex and well-regulated process that involves sympathetic vasoconstrictor signaling in combination with an integration of a large number of vasodilator systems as well as several modes of activation of these systems. To “defend blood pressure” during significant vasodilatation in working muscles, the increased sympathetic activity maintaining the increased CO also causes arteriolar constriction in relatively less active tissues (Joyner and Casey, 2015). Hence, during exercise the increased sympathetic activity causes arteriolar constriction in tissues with low metabolic demand and in the active muscle effective amounts of vasodilators have to be formed to overcome and exceed this constriction to enable sufficient oxygen delivery. In the active muscle, the increase in sympathetic vasoconstriction is overcome by local formation of a number of different vasodilators and by “functional sympatholysis”, a modulation of the vasoconstrictive effect of the sympathetic activity. Our understanding of precisely how skeletal muscle blood flow is regulated in response to changes in metabolic demand is not complete. However, fundamentally it is highly probable that there are signals that are closely coupled to a, yet to be identified, mechanism of oxygen metabolic sensing within the muscle tissue. One such potential mechanism is erythrocyte mediated vasodilation where erythrocytes release the vasodilators ATP (Ellsworth and Sprague, 2012) and nitric oxide (Singel and Stamler, 2005) in response to oxygen desaturation of the hemoglobin molecule, thus connecting the utilization of oxygen by the muscle to the magnitude of blood flow. A number of other mechanisms and compounds, not directly coupled to oxygen sensing or metabolism, have been identified but their relative importance and roles remain unclear. Out of the vasodilatory mechanisms and compounds identified, several induce their effect via sensors or receptors located on the endothelium resulting in the formation of vasodilators. Such endothelial-dependent mechanisms include flow (shear-stress)-induced vasodilatation and vasodilatation balanced by chemicals such as nitric oxide, adenosine triphoshate, adenosine, reactive oxygen species, prostaglandins, endothelial-derived hyperpolarizing factors, and Endothelin-1. Shear stress acutely increases nitric oxide formation (Rubanyi et al., 1986) and upregulates endothelial nitric oxide synthase (Woodman et al., 1999; Tuttle et al., 2001; Baum et al., 2004). Apart from endothelial cells, cellular sources of vasodilator compounds during exercise include skeletal muscle cells, and notably adenosin, ATP, prostacyclin and NO have been suggested to induce vasodilation by direct vasorelaxation of smooth muscle cells (Hellsten et al., 1998; Hellsten et al., 2012b). Of interest, exercise improves flow-mediated vasodilation not only in the exercised limbs but also in non-active limbs (Birk et al., 2012). Another mechanism involved in the control of blood flow to working muscles and which particularly serves to coordinate flow is conducted, or retrograde, vasodilation. In this process a vasoactive signal initiated either at the capillary level (Murrant and Sarelius, 2000) or at the arteriolar level (Segal and Duling, 1989; Bagher and Segal, 2011) is conveyed up and downstream via gap junctions either in the endothelium or in the smooth muscle cells. Some vasodilation may be through the myogenic response by which vessel wall myocytes respond to acute changes in transmural pressure (de Witt et al., 1998). So far it is known that all of these can mechanisms induce vasodilation of importance during exercise (Casey et al., 2013), but none of these seem to be obligatory since a great degree of redundancy exist in that other vasodilator systems take over if one system is pharmacologically inhibited.

### Patients with T2DM

In pts. with hypertension and DM, the blood flow increment during submaximal exercise is often reduced (Kivelä et al., 2006; Thaning et al., 2010; 2011; Sacre et al., 2015; Olver and Laughlin, 2016). Lower arm hyperemia induced by submaximal and maximal hand-grip exercise is reduced in pts. with DM, who also have microvascular complications (Womack et al., 2009). One frequently observed outcome of endurance training, that likely affects the magnitude of blood flow to the muscle during submaximal exercise, is an increased arterio-venous oxygen extraction, which in part occurs as a result of an increased capillarization (Mortensen et al., 2017) and thereby improved oxygen diffusion capacity but which also is dependent on mitochondrial capacity. In a study by Nyberg et al. (2012b), it was found that blood flow during submaximal exercise performed at the same absolute workload was reduced after training in normotensive subjects, indicating an improved oxygen extraction, whereas blood flow was unaltered in hypertensive subjects after 8 weeks of endurance training. The leg vasodilator response to exercise has been reported to be lower in T2DM (Thaning et al., 2010; Groen et al., 2019) although a preserved response has also been demonstrated (Thaning et al., 2011).

Flow-mediated vasodilation is significantly impaired in pts. with DM, and while it is well-proven that exercise training improves this physiological response, it seems that the improvements seen with exercise training in pts. with T2DM are smaller than in non-diabetic controls (Qiu et al., 2018). In pts. with T2DM, training improves arterial flow-mediated (endothelial dependent) and non-endothelial dependent dilatation (Schreuder et al., 2015), and training lowers the skeletal muscle amount of endothelial nitric oxide uncoupling, suggesting enhanced capacity for nitric oxide formation, both in normal subjects and in pts. with hypertension (Nyberg et al., 2012b). Exercise training, especially in the elderly, may enhance nitric oxide availability by reducing scavenging of nitric oxide by reactive oxygen species (Taddei et al., 2000; Franzoni et al., 2005; Kirby et al., 2009; Crecelius et al., 2010; Nyberg et al., 2012b; Nyberg et al., 2014). Thus, training will improve nitric oxide availability partly by increased formation of nitric oxide itself, partly by reduced scavenging of nitric oxide, and it seems training is more important in subjects where it is already impaired. Prostaglandins may contribute to exercise-related vasodilation, and training in pts. with hypertension enhances the increase in muscle interstitial prostacyclin (Hellsten et al., 2012a). Other important vasodilator systems include ATP and adenosine as individuals with T2DM have been reported to have lower plasma ATP concentrations during exercise that occurs in parallel with a lower blood flow and the inhibition of adenosine receptors reduces exercising muscle blood flow by 15%–20% at least in healthy individuals (Radegran and Calbet, 2001; Mortensen et al., 2009). Lifelong physical activity opposes an age-related increase in skeletal muscle and plasma levels of the potent vasoconstrictor endothelin-1, and 8 weeks of training normalizes plasma endothelin-1 levels in individuals with essential hypertension (Nyberg et al., 2013). Furthermore, endothelin-1, is lowered by 30% by training in pts. with hypertension, where training improves not only the levels of endothelin-1 but concomitantly also carotid artery compliance (Maeda et al., 2009). Among vasoconstrictors, angiotensin II is well-known to be associated with vascular dysfunction and has been shown to decrease in response to exercise training (Zucker et al., 2015).

## Resistance arteries and microvascular network of skeletal muscles

### Normal subjects

Capillary density in muscle is important because a small microvascular network of the skeletal muscles limit the ability of an enhanced cardiac output during exercise to supply skeletal muscles with a high blood flow without a reduction in the time for gas exchange at the capillary site. A high vasodilatory capacity combined with a high skeletal muscle capillary volume are thus advantageous adaptations to training. Thus, training increases the vasodilator capacity as evidenced in athletes where a greater peak vasodilator response is seen in the dominant arm of tennis players (Sinoway et al., 1986) and a maximal vasodilator response has been described after a period of forearm training in previously untrained (Sinoway et al., 1987). Moreover, as little as 4 weeks of training can increase capillary density (Jensen et al., 2004) and when previously sedentary healthy individuals are endurance-trained for 4–8 weeks an increase in capillarization of up to 40% is observed (Hoier and Hellsten, 2014; Ingjer, 1979a; Ingjer, 1979b; Klausen et al., 1981). The physiological signals governing capillary growth are still not completely understood but generally involves mechanical stimuli (Hellsten et al., 2008) with both shear stress (Langille and O´Donnell, 1986; Pipp et al., 2004; Tuttle et al., 2001) and passive stretch of the muscle tipping the weight from anti-angiogenic compounds towards pro-angiogenic compounds (Hudlicka and Egginton, 1992). Shear stress is known to be a particularly important physical factor for both arterial and capillary growth where it induces its effect by activating and enhancing the expression of several angiogenic factors, including the central growth factor vascular endothelial growth factor (VEGF), (Hudlicka and Egginton, 1992; Lloyd et al., 2005). Hypoxia, via hypoxia inducible factor 1-alpha is also known to promote capillary growth, in part via transcription of VEGF (Gustafsson et al., 2007). There is a clear correlation between training status and expression of many pro-angiogenic compounds, and studies show increments of these with training in sedentary individual (Gavin et al., 2007; Hoier et al., 2012; Gliemann et al., 2014) including in pts. with primary hypertension (Hansen et al., 2010). Although specifically VEGF protein levels in muscle are not commonly altered by exercise training in young subjects, individuals.

### Patients with T2DM

Training increases the capillary/fibre ratio also in T2D (Lithell et al., 1985), but an attenuation in the exercise-induced increase in capillarization has also been reported in type 1 DM mice in part likely due to reduced levels of proangiogenic factors (VEGF-A, VEGF-B, neuropilin-1, VEGFR-1, and VEGFR-2) and increased levels of antiangiogenic factors (thrombospondin-1 and retinoblastoma like-2) in comparison with normal mice (Kivelä et al., 2006). Thus, exercise training in pts. with DM alleviated some of these changes, but may not completely restore them. T2DM alters capillary hemodynamics, causes capillary rarefaction in skeletal muscle as is seen in the myocardium, and alters endothelial and vascular smooth muscle cell phenotype, resulting in impaired vasodilatory responses (Olver and Laughlin, 2016). Training will improve the endothelial-dependent as well as the non-endothelial-dependent vasodilation in pts. with T2DM (Maiorana et al., 2001). Individuals with T2DM have lower plasma ATP concentrations during exercise and hypoxia compared with control individuals, and this occurs in parallel with lower blood flow. Moreover, individuals with T2DM have a reduced vasodilatory response to infused ATP (Groen et al., 2019). In patients with DM the skeletal muscle vasodilatory effect of purines is only 50% of what is seen in normal subjects, probably as a result of receptor insensitivity (Thaning et al., 2010).

The basement membrane surrounding capillaries in skeletal muscles varies physiologically in thickness according to age, physical fitness, and anatomical site in humans and prematurely thickens with DM (Baum and Bigler, 2016). A thickened capillary basement membrane likely poses a greater barrier for diffusion, lowers the microvascular elasticity, and impedes transcytosis of inflammatory cells of possible importance for among others wound healing in DM (Baum and Bigler, 2016). Interestingly, the basement membrane thickness of capillaries in skeletal muscle has been shown to be reduced by exercise training both in pts. with T2DM (Mortensen et al., 2019) and essential hypertension (Gliemann et al., 2015). As is seen in the myocardium, related to this, T2DM is characterized by a fibrotic extracellular matrix with increased hyaluron and integrins: this may contributs to impaired glucose uptake by skeletal muscle cells and presumably also to oxygen (Hulett et al., 2022), and fibrosis is also seen in skeletal muscles of pts. with DM (Farup et al., 2021). Probably related to the same mechanisms, with long-standing DM even infarctions probably secondary to vascular rarefaction may develop (Trujillo-Santos, 2003).

Preclinical studies and clinical trials involving the use of GLP-1 receptor agonists have shown salutary cardiovascular effects and improved cardiovascular outcomes in T2DM (Love et al., 2020) but the precise impact of GLP-1 receptor agonists and SGLT2i on skeletal and myocardial capillary density and function remain unclear. GLP-1, in addition to its well-characterized glycemic actions, however, improves endothelial function, increases muscle microvascular perfusion, and stimulates angiogenesis (Love et al., 2020). Importantly, these actions are preserved in the insulin resistant states (Love et al., 2020). Thus, treatment of insulin resistant pts. with GLP-1 receptor agonists may improve skeletal and cardiac muscle microvascular perfusion and increase muscle capillarization, leading to improved delivery of oxygen, nutrients, and hormones such as insulin to the myocytes (Love et al., 2020).

## Conclusion

While the pathophysiology driving the emerging “HFpEF epidemic” in pts. with T2DM is heavily influenced by the documented cardiac changes of myocardial hypoperfusion and fibrosis, the peripheral vascular changes are equally important and should be considered when evaluating poor exercise capacity. Thus, T2DM impacts all major cardiovascular mechanisms well-documented to be of importance in exercise and hence for the individual´s ability to lead an active healthy life. In short, documented cardiovascular changes in pts. with T2DM collectively point to a “low-CO-with-exercise-syndrome” that with respect to cardiac and peripheral changes are the antithesis to what is seen with an active healthy lifestyle. Initially, an impaired muscle perfusion with exercise may be (partly) reversed with weight-loss and training since a number of the vasodilatory mechanisms affected seem resilient, but with long-standing T2DM more profound changes such as myocardial and skeletal muscle vascular rarefaction with perivascular fibrosis will limit exercise capacity. In addition to weight-loss by gastric by-pass or treatment with GLP-1 receptor agonists, it has long been well-documented that exercise will reverse most aspects of T2DM and should be added to the weight-loss intervention. Most important aspects of exercise tolerance can be improved by exercise and indeed by weight-loss, but once more substantial cardiac and peripheral changes, such as vascular rarefaction and fibrosis, have occurred, it will be much more difficult to reverse the exercise intolerance. GLP1 receptor agonists and SGLT2 inhibitors may not only to some degree relieve symptoms of frank HF but instituted early may even halt and possibly reverse findings of fibrosis and vascular rarefaction. To combat the epidemic of HFpEF in diabesity, screening with early identification of the underlying pathophysiological changes should be instituted early in order to enable personalized treatment of deeper phenotypical traits all documented to be of importance for difficult-to-reverse cardiovascular changes. Recent epidemiological studies show that the prevalence of T2DM continues to increase worldwide and now also increases substantially in the young (GBD Diabetes Collaborator, 2023), and hence the epidemic of HFpEF will not be confined to the elderly but will probably increase substantially also in the young with potentially a large number of active patient years to protect.
